# Surface Modification to Improve Properties of Materials

**DOI:** 10.3390/ma12030441

**Published:** 2019-01-31

**Authors:** Miran Mozetič

**Affiliations:** Department of Surface Engineering, Jozef Stefan Institute, Jamova cesta 39, 1000 Ljubljana, Slovenia; miran.mozetic@guest.arnes.si

**Keywords:** surface properties, nanostructuring, functionalization, grafting

## Abstract

Surface properties of modern materials are usually inadequate in terms of wettability, adhesion properties, biocompatibility etc., so they should be modified prior to application or any further processing such as coating with functional materials. Both the morphological properties and chemical structure/composition should be modified in order to obtain a desired surface finish. Various treatment procedures have been employed, and many are based on the application of non-equilibrium gaseous media, especially gaseous plasma. Although such treatments have been studied extensively in past decades and actually commercialized, the exact mechanisms of interaction between reactive gaseous species and solid materials is still inadequately understood. This special issue provides recent trends in nanostructuring and functionalization of solid materials with the goal of improving their functional properties.

## 1. Introduction

An important property of solid materials is surface morphology. The morphology governs the effective surface area which is always larger than the macroscopic geometrical area. Most techniques employed in mass production of materials, such as casting, injection molding, extrusion and rolling, enable limited abilities for increasing the surface area well above the geometric one. Solid materials are often prepared from liquids whose surface energy facilitates smooth surfaces. Even upon solidification, such a smooth surface is often preserved. In numerous applications, however, a solid material of a large surface area (much larger than the geometric one) is preferred. A rough surface often enables better adhesion of a coating and is essential in numerous applications. The surface area is usually increased well over the geometric one either by etching or deposition. Etching is usually performed using liquid or gaseous media. Etching by liquid media is often a fast process and thus beneficial in industrial applications, but it has drawbacks, such as inadequate morphology and ecological concerns due to the production of large quantities of waste chemicals. The dry gaseous etching is often much slower but is usually ecologically sustainable and provides almost arbitrary surface morphology. Etching of heterogeneous materials containing different components results in increased surface area due to preferential removal of one component. Homogeneous materials, however, assume rich morphology upon etching due to various mechanisms, including preferential removal of a material at grain boundaries, electrochemical effects, kinetic effects due to bombardment with ions, and self-assembly. The latter is particularly beneficial but limited to specific materials. A trivial method for increasing surface morphology in a highly controlled manner is the deposition of a mask followed by a suitable etching procedure. The etching using a mask is often anisotropic, in particular when the technology of reactive ion etching is applied. An alternative to etching is deposition of an arbitrary material. This technique enables almost arbitrary surface finish.

Apart from morphology, the functional properties of materials are governed by the composition and structure of the surface film. These are always different from the bulk counterparts because of the simple reason that unlike the bulk, where the atoms are surrounded by other atoms in all directions, the surface atoms are bonded to other atoms only from one side. The surface atoms therefore feel the attractive force of other atoms only from one side resulting in the surface energy. Furthermore, the gas-phase atoms or molecules feel the attractive force of surface atoms; therefore, most materials are covered with foreign molecules in practical cases. The surface of most metals and alloys, for example, are oxidized and covered with a thin film of organic molecules that come to the surface from the surrounding air. The composition of the surface is thus usually different from the composition of the bulk material. The ability for bonding foreign atoms and molecules is beneficial in cases where the surface functional properties are inadequate. For example, the surface properties of polymers are tailored rather arbitrarily by functionalization with specific functional groups. These functional groups allow for almost arbitrary wettability of the solid materials. A widely used technique for surface functionalization is a brief treatment with non-equilibrium gaseous plasma.

## 2. This Special Issue 

Scientific papers selected for this issue deal with modification of surface properties of various materials by advanced techniques. Asadollahi et al. [1] describe a method for synthesis of stable super-hydrophobic surfaces through an economical and practical process by development of an organosilicon-based coating using an atmospheric-pressure plasma jet technique. Such a surface finish emerged from the investigation of natural surfaces with a high contact angle often known as the lotus leaf effect [2]. The superhydrophobic characteristics of the micro-nanostructured and wax-coated surface of the lotus leaf was first studied by Dettre and Johnson in 1964 [3]. Since then, numerous authors studied methods for creating such a surface finish. Asadollahi et al. [1] developed a technique using atmospheric pressure plasma polymerization of hexamethyl disiloxane in the jet of nitrogen plasma produced by a rotating arc discharge. The plasma jet was modified by mounting a quartz tube on the jet head, thus confining the plasma jet in a smaller volume, which was found to be beneficial for deposition of high-quality coatings. 

Porous silicon has been found useful in a broad range of industries [4]. Numerous methods for synthesizing such materials have been reported, and the review paper by Lee et al. summarizes breakthroughs and recent trends [5]. Despite extensive work, there is still a great demand for further development of advanced surface modification methods for this material. Recently developed techniques include hydrolytic condensation, ring-opening click chemistry, and synthesis of calcium or magnesium silicates. The next-generation of surface modification methods forecasted in this review [5] will focus on reproducibility suitable for mass application in industry, improved bio-applicability and low toxicity, methods for preventing pore collapse, and multi-functional surface finish. 

An assembly of organosilanes on porous silicon using visible light is reported by Rodriguez et al. [6]. The functionalization of semiconductor nanostructures with organic monolayers is regarded as essential for tailoring the surface chemistry for bioconjugation [7]. Porous silicon is often a matrix of silicon quantum dots immersed into amorphous network containing silica and silicon. It is different from classical nanodots in amorphous silicon networks because of extremely high surface area [8]. Rodriguez et al. employed a condensation process to synthesize organosilane functionalized porous silicon films using aminopropyl-triethoxy-silane and perfluorodecyl-triethoxy-silane at low concentrations. Visible light activation promoted surface oxidation of porous silicon, thus stimulating reaction with organosilanes. The process enabled a rather homogeneous surface with no traces of silane-derived colloidal structures, thus making the process useful as a model for the analysis of interfaces between organosilanes and porous silicon.

Aluminum alloys should be subjected to various postproduction treatments in order to assure required functional properties [9]. An optimal surface finish is particularly important for alloys subjected to severe weather conditions, such as for aircraft [10]. The surface modifications should inhibit oxidation, promote adhesion of further coatings, or reduce staining. Wet chemical etching enables production of porous textured surfaces of aluminum-alloys [11]. Not only an appropriate combination of chemicals but the order of their applications is crucial to prepare porous surfaces, irrespective from the original roughness.

An alternative method for improving surface properties of alloys is the ultrasonic nanocrystal surface modification process [12]. The technique was used for modification of nickel-rich super-alloy Inconel, which is used in aerospace and nuclear industries [13] due to its excellent properties, excepting fretting wear resistance [14]. A high surface compressive residual stress was obtained by ultrasonification together with increased roughness and hardness. Furthermore, the fretting wear resistance was improved, so the technique may be useful for extending the lifespan of aircraft and nuclear components [12]. The technique was also used for the treatment of tantalum of a rather high purity [15]. The treatment caused both an increase in hardness and induced compressive residual stress. The effect was particularly pronounced at high material temperature upon sonification. A plastically deformed layer with the refined nano-grains was observed and correlated to enhanced wear resistance [15].

Yet another technique suitable for surface modification of alloys is shot-peening. This technique affects a thin surface layer and causes tensile plastic strain, resulting in favorable compressive residual stresses. The technique was used for treatment of aluminum alloy Al 6061-T6 welds [16]. This material is also known for its superior properties in airspace industry. The shot-peening was performed by bombarding the surface with glass shot beads according to aerospace recommended levels. The surface morphology was modified upon the treatment, and lower hardness was obtained as compared to the base metal alloy in the heat-affected zone even after short shot-peening treatments [16]. 

A review of methods for surface texturing of the most commonly used titanium alloy (Ti_6_Al_4_V) was also published in this issue [17]. The techniques for achieving appropriate surface morphology of this material include mechanical, electrochemical, and localized heat treatments, in particular laser shock peening, electro spark surface texturing, electrical discharge machining, reactive ion etching, lithography, abrasive jet machining, and anodization. The surface texturing is beneficial in different applications, from self-lubrication of special tools [18] to vascular stents [19].

Surface properties of metals and alloys can be also tailored by the deposition of different coatings. Of particular importance are hard coatings, as demonstrated in Reference [20]. The wear performance increased significantly after depositing nanocomposite coatings with different structures. The films were deposited on aluminum die casting mold tool substrates using gaseous plasma sustained by a pulsed arc discharge. The best results in terms of hardness, soldering behavior, stress, and oxidation resistance were achieved for the AlCrN/Si_3_N_4_ nanocomposite coatings [20]. Such a coating of appropriate thickness enabled improved die-mold service life.

Cemented carbide has been used widely in engineering applications because of a high surface hardness, good thermal stability, outstanding chemical properties, and excellent wear resistance [21]. Calcium fluoride is a widely utilized solid lubricant at high temperatures. The friction coefficient of this lubricant decreases gradually with increasing temperature and exhibits an excellent lubricating effect even at 1000 °C [22]. The performances of microhole-textured carbide tools filled with CaF_2_ was elaborated by Song et al. [23]. They found such materials suitable for promoting machining performance. The textured carbide improved the tribological performance compared to the non-textured material at high machining speed. 

An appropriate surface finish is also important for the development of paper with specific functional properties. In many applications, the paper should exhibit antibacterial properties [24]; therefore, it should be coated with antibacterial nanoparticles. Such nanoparticles do not attach to the cellulose surface unless an appropriate plasma treatment is employed [25]. Schlemmer et al. [26] report a reliable, fast, and eco-friendly method to fabricate paper fines sheets impregnated with silver nanoparticles, which are known for their antimicrobial activity. The standard route for paper sheet formation was modified with an additional step using colloidal nanoparticles. The key observation reported in this paper is that a highly stable nanoparticle solution is essential for good dispersion of nanoparticles into a paper solution, and thus reasonably homogeneous distribution in the final product.

Surface properties of organic materials are also important in the development of alternative drugs, in particular for curing cancer diseases. Promising materials include lectins, which are important for cell communication and signaling in many physiologic as well as pathophysiologic processes [27]. Recently, it was reported that lectin interactions with tumor-specific glycan epitopes promote tumor growth and immune modulation [28]. A critical issue is adsorption of lectins onto suitable substrates, as reported by Niegelhell et al. [29]. They found the largest adsorbed amounts and the fastest adsorption kinetics on the surface of polystyrene. The adsorption kinetics of the examined lectins were found comparable to bovine serum albumin. The polysaccharide layers are also found to be prone to swelling.

The modification of polymer materials by non-equilibrium gaseous plasma has attracted enormous attention in the past decades due to industrial demands. Recent advances in the complex phenomena occurring upon interaction of reactive gaseous species with polymer surfaces have been summarized in an extensive review [30]. Particularly important are fluorinated polymers such as Teflon due to broad applications from kitchenware to medicine. Surface functionalization of this material is challenging, especially when exotic functional groups should be grafted to mimic biomaterials [31]. The paper by Lopez-Garcia et al. [32] reports morphological and structural modifications of Teflon surfaces upon treatment with non-equilibrium plasma sustained by different discharges. The treatment resulted in the modification of surface morphology, whereas functionalization with polar groups was found to be moderate even though a variety of treatment parameters were tested in this paper.

A broad application of polymers occurs in food packaging. The demand for packed food is increasing rapidly, but a major obstacle is the life span of fresh products. The polymer foils suitable for mass application in food packaging lack antimicrobial properties and are rather permeable by oxygen; therefore, they should be coated with suitable coatings. Due to the hydrophobic character of standard foils, the adhesion of any coating should be improved, and a natural choice is the application of gaseous plasma [33]. The plasma parameters, however, should be chosen carefully to prevent any damage to other foil properties which might result from VUV radiation from gaseous plasma [34]. An alternative technique which prevents such effects is the application of extremely short treatments in weak plasma where radiation is almost absent, but the concentration of neutral reactive species are still comparable to that in ordinary plasma. The efficiency of such an approach was demonstrated in paper [35]. 

Polymer composites are among the most widely used materials. Their properties depend on the type of polymer blends and fillers, as well as the distribution and even orientation of fillers in the polymer matrix [36]. The fillers tend to agglomerate in the polymer matrix, which is particularly important for two-dimensional carbon materials such as graphene [37]. Researchers worldwide are developing novel methods for the modification of fillers’ surface properties in order to obtain the desired quality of products. Of particular important are biofillers extracted from natural sources. Shah et al. [38] elaborated the modification of egg shell particles using stearic acid and their reinforcement in the epoxy-polymer matrix. They report excellent toughness, elongation increase, and reduced brittleness of such composites.

Antimicrobial properties, biodegradability, and biocompatibility are important properties for polymer materials used in food packaging, medical, and pharmaceutical applications, as well as in cosmetics. Such properties of polymers could be achieved by appropriate immobilization of active coatings onto a polymer surface, but a serious obstacle is often poor adhesion due to the inadequate wettability, which should be modified [39]. Antimicrobial agents can be incorporated directly into polymers, or they are attached via the side chains. Polyvinyl alcohol (PVA) substrates were treated with plasma created by coplanar surface barrier atmospheric pressure discharge and cross-linked with glutaric acid. Such a pretreatment allowed for optimal nisin adhesion [40].

While polymer foils or plastic components are rather quickly activated using appropriate plasma parameters, the technology is more demanding in cases where small granules should be treated. This topic was addressed by Šourkova et al. [41]. The authors used a low pressure plasma reactor powered with a pulsed microwave discharge with a stirring devise inserted into the plasma reactor. Such an experimental configuration allowed for a low density of charged particles in gaseous plasma, but the density of neutral oxygen atoms was as high as 2 × 10^21^ m^−3^ in a large volume at a relatively low discharge power density. As a result, the polyethylene granules were effectively functionalized with polar groups without measurable etching of the polymer material. Such rapid functionalization is a consequence of a very large affinity of the polymer surface to atomic oxygen [42].

While functionalization with polar groups is beneficial for adhesion of various coatings on polymer materials, there are applications where the surface finish should prevent sticking of unwanted liquids to the polymer surface. In such cases, the polymer should be functionalized with non-polar groups, and the best are fluorine-containing groups. Several fluorine-containing gases dissociate to F atoms under plasma conditions, but the surface finish depends on the type of the precursor. Resnik et al. [43] compared plasma treatment of polyethylene terephthalate using two gases and reveal effects that have not been observed before. The treatment of this polymer with plasma sustained both in tetrafluoromethane and sulfur hexafluoride revealed a high concentration of fluorine in the surface layer probed by X-ray photoelectron spectroscopy (XPS) only up to a certain pressure. Thereafter, both the fluorine content and the water contact angle decreased significantly, which was explained by the lack of F atoms at elevated pressure using low power density plasma.

Biomimetics is a hot topic in interdisciplinary materials science. A review paper has been published on various examples of successful synthesis and application of materials mimicking nature [44]. The paper describes wetting properties of man-made materials mimicking both super-hydrophobic and super-hydrophilic surfaces of plants and animals, with a particular emphasis of combining both effects on a scale measured in micrometers. Such materials have extremely high potential for application for body implants because the adsorption and conformation of proteins depends enormously on the surface wettability. The activation of blood platelets on such surfaces is reduced significantly; therefore, such a surface finish represents an alternative to biomaterials of laterally uniform surface finish [45,46]. 

Hemo-compatibility of body implants made from polymeric materials has attracted enormous attention in the past decades due to medical applications. Currently, cardiovascular diseases represent the main causes of mortality in the modern world. Treatments include implanting stents, artificial heart valves, and vascular grafts. An ideal surface finish of such implants should not only prevent activation of blood platelets, but also inhibit scar formation and facilitate rapid endothelization. These requirements are contradictory; therefore, researchers worldwide are investigating methods for producing such a surface finish that would meet all requirements. A review paper on recent advances in biocompatibility of plasma-treated polymeric implants was prepared by Recek [47]. An extensive literature review led to the conclusion that there are actually no available standardized methods for testing the hemocompatibility of biomaterials. In this review paper, the most promising methods to gain biocompatibility of synthetic materials are reported, and several hypotheses to explain the improvement in hemocompatibility of plasma treated polymer materials are offered.

Mushrooms represent an important nutritional component of the human diet and are valuable in many countries; therefore, the demand often exceeds the supply. A method for increased growth employs treatment of the substrates by pulsed electrical fields [48]. Several power supplies have been tested for the promotion of mushroom growth by electrical stimulation reaching voltages on the order of 100 kV [49]. The efficiency of such treatments depended enormously on the type of mushroom; therefore, an appropriate voltage has to be adopted according to particular conditions [39]. Unlike conventional methods, the innovative power supply reported in this issue by Takahashi et al. [48] enabled application also in hilly and mountainous areas and resulted in a significant improvement in mushroom production. 

## References

[1] Asadollahi S., Profili J., Farzaneh M., Stafford L. (2019). Development of organosilicon-based superhydrophobic coatings through atmospheric pressure plasma polymerization of HMDSO in nitrogen plasma. Materials.

[2] Guo Z., Liu W., Su B.-L. (2011). Superhydrophobic surfaces: From natural to biomimetic to functional. J. Colloid Interface Sci..

[3] Johnson R.E., Dettre R.H. (1964). Contact angle hysteresis. Iii. Study of an idealized heterogeneous surface. J. Phys. Chem..

[4] Sailor M.J. (2012). Porous Silicon in Practice: Preparation, Characterization and Applications.

[5] Lee S.H., Kang J.S., Kim D. (2018). A mini review: Recent advances in surface modification of porous silicon. Materials.

[6] Rodriguez C., Muñoz Noval A., Torres-Costa V., Ceccone G., Manso Silván M. (2019). Visible light assisted organosilane assembly on mesoporous silicon films and particles. Materials.

[7] Sacarescu L., Roman G., Sacarescu G., Simionescu M. (2016). Fluorescence detection system based on silicon quantum dots-polysilane nanocomposites. Express Polym. Lett..

[8] Mäkilä E., Bimbo L.M., Kaasalainen M., Herranz B., Airaksinen A.J., Heinonen M., Kukk E., Hirvonen J., Santos H.A., Salonen J. (2012). Amine modification of thermally carbonized porous silicon with silane coupling chemistry. Langmuir.

[9] Sheasby P.G., Pinner R., Wernick S. (2001). The Surface Treatment and Finishing of Aluminium and Its Alloys.

[10] Heinz A., Haszler A., Keidel C., Moldenhauer S., Benedictus R., Miller W.S. (2000). Recent development in aluminium alloys for aerospace applications. Mater. Sci. Eng. A.

[11] Kadlečková M., Minařík A., Smolka P., Mráček A., Wrzecionko E., Novák L., Musilová L., Gajdošík R. (2018). Preparation of textured surfaces on aluminum-alloy substrates. Materials.

[12] Amanov A., Umarov R., Amanov T. (2018). Increase in strength and fretting resistance of alloy 718 using the surface modification process. Materials.

[13] Vesel A., Drenik A., Elersic K., Mozetic M., Kovac J., Gyergyek T., Stockel J., Varju J., Panek R., Balat-Pichelin M. (2014). Oxidation of Inconel 625 superalloy upon treatment with oxygen or hydrogen plasma at high temperature. Appl. Surf. Sci..

[14] Ott E.A., Groh J.R., Banik A., Dempster I., Gabb T.P., Helmink R., Liu X., Mitchell A., Sjoberg G.P., Wusatowska-Sarnek A. (2012). Superalloy 718 and Derivatives.

[15] Chae J.-M., Lee K.-O., Amanov A. (2018). Gradient nanostructured tantalum by thermal-mechanical ultrasonic impact energy. Materials.

[16] Atieh A.M., Rawashdeh N.A., AlHazaa A.N. (2018). Evaluation of surface roughness by image processing of a shot-peened, TIG-welded aluminum 6061-T6 alloy: An experimental case study. Materials.

[17] Lin N., Li D., Zou J., Xie R., Wang Z., Tang B. (2018). Surface texture-based surface treatments on Ti_6_Al_4_V titanium alloys for tribological and biological applications: A mini review. Materials.

[18] Tang W., Zhou Y.K., Zhu H., Yang H.F. (2013). The effect of surface texturing on reducing the friction and wear of steel under lubricated sliding contact. Appl. Surf. Sci..

[19] Flasker A., Kulkarni M., Mrak-Poljsak K., Junkar I., Cucnik S., Zigon P., Mazare A., Schmuki P., Iglic A., Sodin-Semrl S. (2016). Binding of human coronary artery endothelial cells to plasma-treated titanium dioxide nanotubes of different diameters. J. Biomed. Mater. Res. A.

[20] Paiva J.M., Fox-Rabinovich G., Locks Junior E., Stolf P., Seid Ahmed Y., Matos Martins M., Bork C., Veldhuis S. (2018). Tribological and wear performance of nanocomposite PVD hard coatings deposited on aluminum die casting tool. Materials.

[21] Haubner R., Lessiak M., Pitonak R., Kopf A., Weissenbacher R. (2017). Evolution of conventional hard coatings for its use on cutting tools. Int. J. Refract. Met. Hard Mater..

[22] Deng J.X., Cao T.K., Ding Z.L., Liu J.H., Sun J.L., Zhao J.L. (2006). Tribological behaviors of hot-pressed Al_2_O_3_/TiC ceramic composites with the additions of CaF_2_ solid lubricants. J. Eur. Ceram. Soc..

[23] Song W., Wang S., Lu Y., Xia Z. (2018). Tribological performance of microhole-textured carbide tool filled with CaF_2_. Materials.

[24] Breitwieser D., Spirk S., Fasl H., Ehmann H.M.A., Chemelli A., Reichel V.E., Gspan C., Stana-Kleinschek K., Ribitsch V. (2013). Design of simultaneous antimicrobial and anticoagulant surfaces based on nanoparticles and polysaccharides. J. Mater. Chem. B.

[25] Gorjanc M., Mozetic M., Vesel A., Zaplotnik R. (2018). Natural dyeing and UV protection of plasma treated cotton. Eur. Phys. J. D.

[26] Schlemmer W., Fischer W., Zankel A., Vukušić T., Filipič G., Jurov A., Blažeka D., Goessler W., Bauer W., Spirk S. (2018). Green procedure to manufacture nanoparticle-decorated paper substrates. Materials.

[27] Sze K.L., Tzi B.N. (2011). Lectins: Production and practical applications. Appl. Microbiol. Biotechnol..

[28] Taniguchi N., Kizuka Y., Drake R.R., Ball L.E. (2015). Glycans and Cancer: Role of N-Glycans in Cancer Biomarker, Progression and Metastasis, and Therapeutics. Advances in Cancer Research.

[29] Niegelhell K., Ganner T., Plank H., Jantscher-Krenn E., Spirk S. (2018). Lectins at interfaces—An atomic force microscopy and multi-parameter-surface plasmon resonance study. Materials.

[30] Alenka V., Miran M. (2017). New developments in surface functionalization of polymers using controlled plasma treatments. J. Phys. D Appl. Phys..

[31] Vesel A., Kovac J., Zaplotnik R., Modic M., Mozetic M. (2015). Modification of polytetrafluoroethylene surfaces using H_2_S plasma treatment. Appl. Surf. Sci..

[32] López-García J., Cupessala F., Humpolíček P., Lehocký M. (2018). Physical and morphological changes of poly(tetrafluoroethylene) after using non-thermal plasma-treatments. Materials.

[33] Pankaj S.K., Bueno-Ferrer C., Misra N.N., Milosavljevic V., O’Donnell C.P., Bourke P., Keener K.M., Cullen P.J. (2014). Applications of cold plasma technology in food packaging. Trends Food Sci. Technol..

[34] Zhang Y., Ishikawa K., Mozetič M., Tsutsumi T., Kondo H., Sekine M., Hori M. (2019). Polyethylene terephthalate (PET) surface modification by VUV and neutral active species in remote oxygen or hydrogen plasmas. Plasma Process. Polym..

[35] Vukušić T., Vesel A., Holc M., Ščetar M., Jambrak A.R., Mozetič M. (2018). Modification of physico-chemical properties of acryl-coated polypropylene foils for food packaging by reactive particles from oxygen plasma. Materials.

[36] Sunny A.T., Mozetic M., Primc G., Mathew S., Thomas S. (2017). Tunable morphology and hydrophilicity to epoxy resin from copper oxide nanoparticles. Compos. Sci. Technol..

[37] Huskic M., Bolka S., Vesel A., Mozetic M., Anzlovar A., Vizintin A., Zagar E. (2018). One-step surface modification of graphene oxide and influence of its particle size on the properties of graphene oxide/epoxy resin nanocomposites. Eur. Polym. J..

[38] Shah A.H., Zhang Y., Xu X., Dayo A.Q., Li X., Wang S., Liu W. (2018). Reinforcement of stearic acid treated egg shell particles in epoxy thermosets: Structural, thermal, and mechanical characterization. Materials.

[39] Asadinezhad A., Novák I., Lehocký M., Sedlařík V., Vesel A., Junkar I., Sáha P., Chodák I. (2010). A physicochemical approach to render antibacterial surfaces on plasma-treated medical-grade PVC: Irgasan coating. Plasma Process. Polym..

[40] Kolarova Raskova Z., Stahel P., Sedlarikova J., Musilova L., Stupavska M., Lehocky M. (2018). The effect of plasma pretreatment and cross-linking degree on the physical and antimicrobial properties of nisin-coated PVA films. Materials.

[41] Šourková H., Primc G., Špatenka P. (2018). Surface functionalization of polyethylene granules by treatment with low-pressure air plasma. Materials.

[42] Vesel A., Zaplotnik R., Kovac J., Mozetic M. (2018). Initial stages in functionalization of polystyrene upon treatment with oxygen plasma late flowing afterglow. Plasma Sources Sci. Trans..

[43] Resnik M., Zaplotnik R., Mozetic M., Vesel A. (2018). Comparison of SF_6_ and CF_4_ plasma treatment for surface hydrophobization of pet polymer. Materials.

[44] Avrămescu R.-E., Ghica M.V., Dinu-Pîrvu C., Prisada R., Popa L. (2018). Superhydrophobic natural and artificial surfaces—A structural approach. Materials.

[45] Sopotnik M., Leonardi A., Krizaj I., Dusak P., Makovec D., Mesaric T., Ulrih N.P., Junkar I., Sepcic K., Drobne D. (2015). Comparative study of serum protein binding to three different carbon-based nanomaterials. Carbon.

[46] Humpolicek P., Kucekova Z., Kasparkova V., Pelkova J., Modic M., Junkar I., Trchova M., Bober P., Stejskal J., Lehocky M. (2015). Blood coagulation and platelet adhesion on polyaniline films. Colloid. Surf. B.

[47] Recek N. (2019). Biocompatibility of plasma-treated polymeric implants. Materials.

[48] Takahashi K., Miyamoto K., Takaki K., Takahashi K. (2018). Development of compact high-voltage power supply for stimulation to promote fruiting body formation in mushroom cultivation. Materials.

[49] Takaki K., Yoshida K., Saito T., Kusaka T., Yamaguchi R., Takahashi K., Sakamoto Y. (2014). Effect of electrical stimulation on fruit body formation in cultivating mushrooms. Microorganisms.

